# Transboundary Dispersal Dynamics of *Ceracris kiangsu*: From Source Regions to Migration Corridors

**DOI:** 10.3390/insects16040400

**Published:** 2025-04-11

**Authors:** Yangyang Li, Ting Du, Jun Yao, Yunsen Chen, Lei Shi, Sangzi Ze

**Affiliations:** 1 Key Laboratory of Breeding and Utilization of Resource Insects of National Forestry and Grassland Administration, Institute of Highland Forest Science, Chinese Academy of Forestry, Kunming 650224, China; lyangyang304@gmail.com (Y.L.); duting_x_y@163.com (T.D.); eyaojun@hotmail.com (J.Y.); 2 Yunnan Key Laboratory of Breeding and Utilization of Resource Insects, Kunming 650224, China; 3 Graduate School, Nanjing Forestry University, Nanjing 210037, China; 4 Yunnan Academy of Agricultural Engineering, Kunming 650216, China; 15987161680@163.com; 5 Yunnan Forestry and Grassland Pest Control and Quarantine Bureau, Kunming 650051, China

**Keywords:** *Ceracris kiangsu*, trajectory analysis, source regions, migration corridors

## Abstract

Yunnan is located on the southwest border of China, with a complex geographical environment and rich biodiversity, which is the first stop for many migratory pests to enter China. In recent years, *Ceracris kiangsu* has migrated into China through the China–Laos border line. The migratory *C. kiangsu* has shown typical characteristics of migratory locusts, which has seriously jeopardized the ecological security, biosecurity and food security of China. In order to prevent and control *C. kiangsu* from the source as soon as possible, this study used hotspot analysis and trajectory analysis to clarify the migration dynamics, source regions and migration corridors of *C. kiangsu*. The results showed that the migratory *C. kiangsu* was mainly distributed in the towns of Jiangcheng County, and the source regions were concentrated in Phongsaly, Laos. There are three cross-border migration corridors of *C. kiangsu*, among which the Laos–Niuluohe border migration corridor running through the entire migration cycle is the most important corridor. The study answered three key questions about the prevention and control of *C. kiangsu*. Ascertaining when *C. kiangsu* arrived at Yunnan, where it came from, and where the population then went will greatly improve the efficiency of the prevention and control of *C. kiangsu* as well as provide a theoretical basis for subsequent monitoring and early warning.

## Simple Summary

*Ceracris kiangsu*, a migratory phytophagous pest of significant agroforestry concern, has recently been identified as a priority target for prevention and control in Yunnan Province, China. Persistent transboundary incursions from Laos via the China–Laos border regions have escalated the risks to local agroecosystems and forested landscapes. However, key uncertainties persist regarding the geographic origins of these migratory populations, their phenological migration patterns, and their spatiotemporal dispersal corridors. To resolve these questions, we integrated geospatial hotspot analysis and trajectory modeling to reconstruct the dynamics of source regions and migratory pathways. Our results demonstrate that the source regions are largely confined to northern Laos with three predominant migration corridors exhibiting distinct spatiotemporal characteristics. The migration window extends from late June to early August with peak activity being observed in July. Notably, the Laos–Niuluohe border migration corridor emerges as the predominant corridor.

## 1. Introduction

*Ceracris kiangsu* Tsai 1929, belonging to Orthoptera Acrididae, is an omnivorous migratory pest [1], which mainly harms bamboos, including *Thysanolaena maxima*, *Trachycarpus fortunei*, and *Musa basjoo* Siebold, as well as crops such as maize, rice, millet, and sorghum [2,3]. *C. kiangsu* is distributed across India, Thailand, Laos, and southern China with an annual single generation [3]. It overwinters as eggs deposited in egg pods 1–2 cm beneath the soil surface. The hatching time of overwintering eggs varies geographically, occurring from mid-April in northern regions to early June in southern latitudes. The nymphal stage comprises five instars over approximately two months [4,5,6]. Adults attain mating capability 10 days after emergence, which is followed by a requisite 10-day feeding period prior to oviposition. Gravid females preferentially oviposit in loose, sparsely vegetated soils adjacent to bamboo forests, producing one to six egg pods throughout their lifespan, each containing 20–35 eggs. Females die within 5–7 days after oviposition [7]. Due to *C. kiangsu* being a large feeder, long-lasting pest, it is difficult to control [8]. Nearly 6000 hectares of farmland in Hunan, China, were infested in 1946, resulting in crop losses valued at approximately CNY 3 million. Since 2015, recurrent outbreaks in Laos have severely impacted bamboo forests and gramineous crops (e.g., rice and maize) [9]. In 2020, large-scale migrations caused extensive agricultural damage in Laos and Vietnam, followed by incursions into Yunnan, China, where over 10,000 hectares of agroforestry land were infested. Localized population densities reached epidemic levels of 200–800 individuals/m^2^ [10], leading to severe ecological and economic consequences.

Early studies concluded that *C. kiangsu* could not migrate over long distances [11], and its destructive power was limited, so there were few studies on the migration of *C. kiangsu*. However, the migratory events in recent years have shown that *C. kiangsu* can migrate tens of kilometers or nearly 100 km at a time [12], and the species can migrate many times. The monitoring data show that *C. kiangsu* along the border between China and Laos has a straight-line distance of 235 km from the border line to the northernmost landing point, which is much further than the traditional belief that *C. kiangsu* can only migrate 100 km in its lifetime [13]. Therefore, although the flight distance of *C. kiangsu* is not far compared with the flying locust, it also has the ability to migrate a medium—and short—distance.

The long-distance migratory process of insects is the result of the interplay between atmospheric background and motion, and most research on migratory pests draws on atmospheric trajectory analysis methods [14,15,16,17,18]. China, situated within the East Asian Monsoon Zone, serves as a recognized critical migration corridor for aerial insect biomass flux in East Asia [19,20]. Yunnan is influenced by both the Indian monsoon and the East Asian monsoon, and it is also under the control of the atmospheric circulation system of the Tibetan Plateau [18]. Yunnan serves as the southern gateway of China. Its unique climate and geographical position render it one of the principal entry points for insect migration into China. *Nilaparvata lugens* originating from outside China migrates into the southwestern rice-growing regions of Yunnan via the southwest monsoon from northern Thailand and Myanmar [21]; *Spodoptera frugiperda*, a “super pest” native to the Americas, enters China through the Indochina Peninsula and Yunnan, and it can migrate northward as far as the northeast of China [22]; In addition to these, other migratory pests that enter the Chinese mainland through Yunnan include noctuid moths such as *Spodoptera exigua*, *Agrotis ipsilon*, and *Mythimna separata*. Therefore, the border area of Yunnan plays an important role in the management and control of migratory pests.

So, when did *C. kiangsu* arrived in Yunnan, where did they come from, and where did they go? These questions are crucial for the prevention and control of *C. kiangsu* from the source. “Where did they come from” requires clarifying the source region of *C. kiangsu*, and “where did they go” requires tracking their migratory routes. In order to solve these problems, this study, based on the analysis of the migratory dynamics of *C. kiangsu*, employs trajectory analysis methods to trace the source regions of cross-border migratory *C. kiangsu* and explore their migratory corridors so as to provide scientific and accurate prevention and control strategies for the prevention and control of *C. kiangsu* from the source.

## 2. Materials and Methods

### 2.1. Data

The study area is located in the region of southeast Asia where *C. kiangsu* cross-border migrates into Yunnan, including five prefectures and cities in the southern and central parts of Yunnan Province. The study area is characterized by significant variations in elevation with a predominantly mountainous and plateau topography. In addition to extensive forest cover, there is a wide range of *C. kiangsu* host plants—bamboos and other graminaceous plants distributed in the area from 300 to 1200 m above sea level [23].

**Figure 1:**
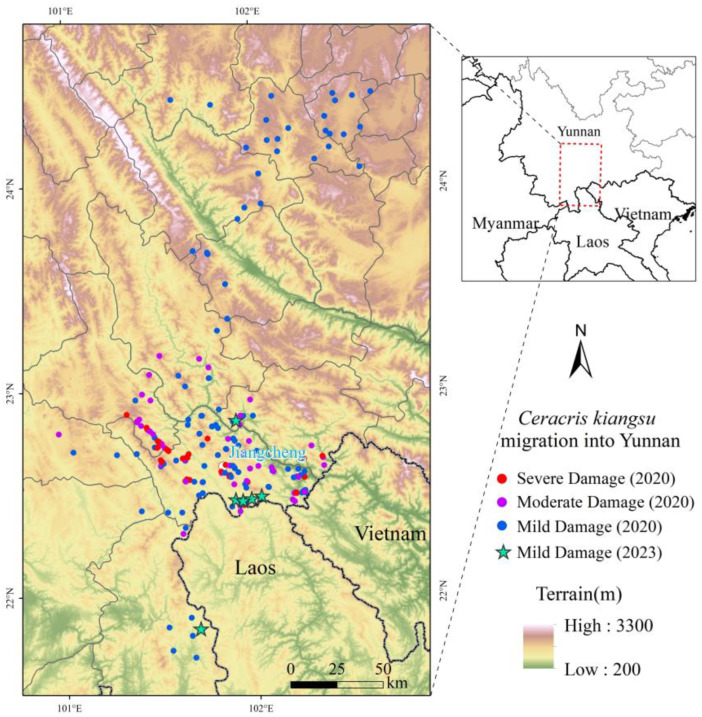
Location of the study area and field survey of *Ceracris kiangsu* migration points. The red, purple, and blue dots represent migration points of *C. kiangsu* in 2020 with severe, moderate, and mild damage, respectively; green stars indicate mild damage points recorded in 2023.

The applied field data were derived from on-site investigations conducted during the migratory period of *C. kiangsu*. The dataset comprises 235 migration points from 2020 and 6 from 2023 (Figure 1), documenting the discovery time, geographic coordinates (latitude/longitude), and severity of damage for each locust migration event. Damage severity was categorized as mild, moderate, or severe based on the classification criteria outlined in Yunnan Province’s *Technical Protocol for Controlling Transboundary Invasions of Ceracris kiangsu* (Table 1) [24]. The vegetation data, which can be used to identify the host plants distribution for *C. kiangsu*, were obtained from the National Tibetan Plateau/Third Pole Environment Data Center (https://data.tpdc.ac.cn) of China [25,26,27]. Meteorological data were obtained from global reanalysis data FNL (Final Operational Global Analysis) provided by the National Centers for Environmental Prediction (NCEP) and the National Center for Atmospheric Research (NCAR). This dataset provides a temporal resolution of 6 h and a spatial resolution of 1° × 1°, and it can be used to simulate the migratory trajectories of *C. kiangsu* and analyze the atmospheric background.

### 2.2. Methods

#### 2.2.1. Detection of Immigration Hotspots for *C. kiangsu*

To identify the hotspots of the migratory *C. kiangsu* populations, we used the Getis-Ord Gi* algorithm to determine the spatial clustering of their distribution. In this study, a total of 241 migratory event records of *C. kiangsu* in 2020 and 2023 were assigned severity-based weights (mild = 1; moderate = 2; severe = 3). After assigning values to the migration points of each township, the values were aggregated. Subsequently, a hotspot analysis was conducted on the cumulative polygonal data of the townships. When significant spatial clustering or the dispersion of *C. kiangsu* migratory populations was detected, significant z-score and *p*-values were generated. These results indicate the presence of spatial hotspots or coldspots in the migratory populations distribution [28]. Regions with *p*-values below 0.05 were identified as significant hotspots. A high z-score in a region indicates a higher density of migratory points of *C. kiangsu* populations compared with neighboring regions. Conversely, when the z-value is close to 0 (with *p* > 0.05), it suggests the absence of a significant spatial aggregation relationship. The study directly invoked the Getis-Ord Gi* algorithm, which has been integrated into ArcGIS 10.3, to calculate the distribution hotspots of *C. kiangsu* populations.

#### 2.2.2. Backward Trajectory Analysis of *C. kiangsu*

The source tracing analysis of *C. kiangsu* includes identifying the source regions during the early migration stage and the peak migration period. We selected the initial detection dates and locations of *C. kiangsu* across different regions to establish starting points for backward tracing early migration trajectories. Based on the identified hotspots and the peak migration period of *C. kiangsu*, we performed a backward trajectory analysis during this phase. This approach allowed the identification of *C. kiangsu* source regions during both the initial migration stage and the peak migration period.

The migration trajectory simulations were performed using the HYSPLIT [29,30,31] (Hybrid Single-Particle Lagrangian Integrated Trajectory) atmospheric particle tracking model (https://www.ready.noaa.gov/HYSPLIT_traj.php, accessed on 12 December 2023), which is a computational platform co-developed by the National Oceanic and Atmospheric Administration (NOAA) of the United States and the Australian Bureau of Meteorology. The migratory trajectory analysis was based on flight behavior parameters of *C. kiangsu* recorded during the 2020 migration period: (1) *C. kiangsu* migrates via air currents. (2) the migratory flights of *C. kiangsu* predominantly occurred on sunny and windy days between 16:00 and 19:30 (Beijing time) with durations exceeding 1 h and extending up to 3.5 h [2]. Based on these observations, the time of take-off was set to 16:00 Beijing time (8:00 UTC), and the time of landing was set to 19:00 Beijing time (11:00 UTC). (3) Flight altitudes were configured at 200 m, 400 m, and 600 m above ground level (AGL) [2]. (4) *C. kiangsu* generally do not fly continuously [2], so our migration simulations assumed a single continuous flight bout. We imported all the trajectories into ArcGIS 10.3, where the simulated trajectories were integrated with spatial distribution data on the host vegetation through an overlay analysis. Valid trajectories were subsequently filtered and visualized to analyze migration pathways and identify potential source regions.

#### 2.2.3. Forward Trajectory Analysis of *C. kiangsu*

Although *C. kiangsu* does not typically exhibit continuous migratory flights, aggregated individuals that have previously landed may re-initiate migratory flights within 2–3 days under conditions of food scarcity or favorable atmospheric dynamics [2]. Based on pest monitoring data, the populations observed in northern Yunnan could not have resulted from a single migratory event. Therefore, representative infestation sites during peak migration periods were selected. By utilizing their geographic coordinates (latitude and longitude) and integrating key migratory behavioral parameters of *C. kiangsu*, daily forward trajectory analyses were conducted. Invalid trajectories coinciding with precipitation events or non-host plants areas were systematically filtered out to refine the re-migration trajectory reconstruction. The analytical methodology for trajectory modeling and the migratory behavioral parameters were maintained consistent with those detailed in Section 2.2.2.

#### 2.2.4. Meteorological Background Analysis

The monsoon is an important factor influencing migratory insects. The migration altitude of *C. kiangsu* has been observed to range between 200 and 600 m above ground, corresponding to the 850 hPa pressure level. Based on the altitude and atmospheric pressure in the migration area, the wind direction and speed at the 850 hPa level were extracted from the FNL data. These data were analyzed using GrADS (Grid Analysis and Display System, version 2.2.1), a meteorological data processing software, to investigate airflow patterns affecting insect migration.

## 3. Results

### 3.1. Hotspots Distribution of Migratory C. kiangsu Populations

**Figure 2:**
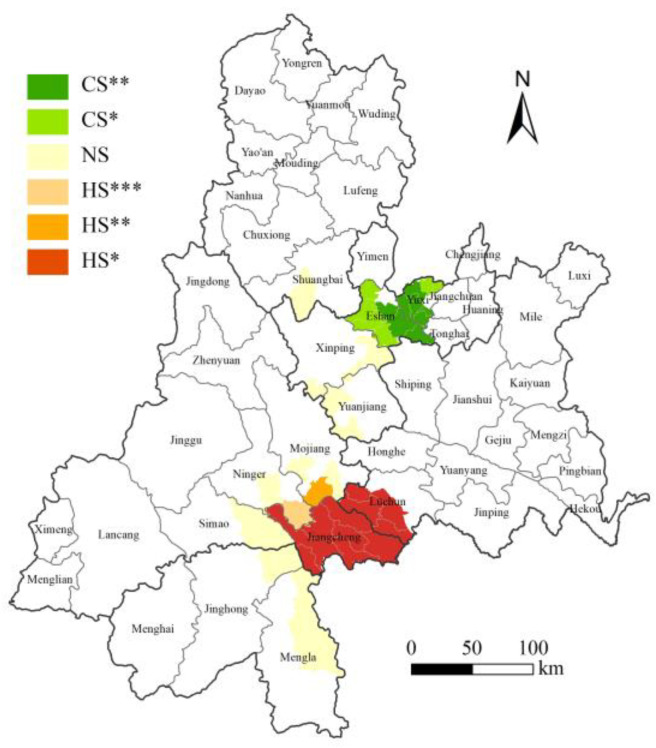
Distribution of hotspots of the migratory *C. kiangsu* populations in 2020 and 2023. HS: hotspots; CS: coldspots; NS: not significant. The distributions marked with *, **, and *** indicate significance levels of 0.1, 0.05, and 0.01, respectively.

The year 2020 marked the peak of *C. kiangsu* migration events and populations abundance since its initial cross-border incursion into China in 2018, which landed in five prefectures and cities in Yunnan Province in batches. A minor migratory population was observed to settle in the China–Laos border area of Jiangcheng County in 2023. Spatially, the migratory populations exhibited distinct distribution patterns (Figure 2): hotspots were concentrated in the township-level jurisdictions of Jiangcheng County and the adjacent southeastern areas of Lüchun County. Coldspots dominated most of the invaded regions of Yuxi City. Other areas showed no significant clustering or dispersion trends based on the Getis-Ord Gi* spatial autocorrelation analysis.

### 3.2. Trajectory and Source Regions in Early Migratory Stage

Based on the collated insect data, the initial observed locations and time of the migration of *C. kiangsu* across various regions in Yunnan Province were determined (Table 2). Our analysis revealed that *C. kiangsu* typically crosses the border into Yunnan in late June with large-scale populations being observed in the counties of Pu’er City during early July. Concurrently, infestations were detected in Mengla County, Xishuangbanna Prefecture in early July. By mid-July, the locusts had migrated to Lüchun County, Honghe Prefecture and multiple counties in Yuxi City. The pests then reached Shuangbai County, Chuxiong Prefecture in early August, exhibiting an overall expansion pattern progressing from south to north movement followed by east-to-west movement. Representative locations from Table 2 were selected as simulation starting points for the backward trajectory analysis to reconstruct the early migration pathways of *C. kiangsu*.

**Figure 3:**
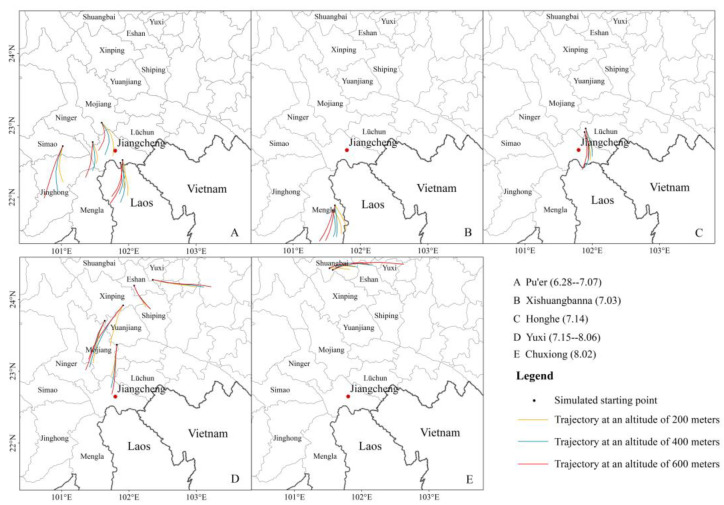
Backward trajectories of *C. kiangsu* in different regions during early migration in 2020. The black dots represent the initial detection locations of the adult, which serve as the starting points for the backward trajectories. (**A**–**E**) Descriptions of the five states (cities) where *C. kiangsu* migrated. The yellow, blue, and red lines indicate the migration trajectories of *C. kiangsu* at altitudes of 200 m, 400 m, and 600 m, respectively.

The earliest cross-border populations of *C. kiangsu* in the China–Laos border area of Pu’er City were identified through backward trajectory simulation analysis. This analysis revealed that locusts invading in late June primarily originated from Phongsaly Province, Laos. In early July, *C. kiangsu* continued migrating northward. The backward trajectories of initial sightings in Ning’er and Mojiang Counties both traced back to Jiangcheng County, indicating that *C. kiangsu* in these two regions migrated from the China–Laos border area. Meanwhile, *C. kiangsu* in Simao likely originated from Xishuangbanna (Figure 3A). The trajectory simulations showed that most locusts in Mengla County, Xishuangbanna, were locally sourced with a minority coming from Laos (Figure 3B).

In mid-July, locusts were detected in Lüchun County with backward trajectories tracing their origin to Jiangcheng County (Figure 3C). Initial sightings in Yuxi persisted from mid-July to early August. Locusts found in Yuanjiang during mid-July were traced back to Mojiang and Jiangcheng counties, while those in Xinping County in late July primarily originated from Yuanjiang (Figure 3D). In Shuangbai County, the backward trajectories (Figure 3E) indicated that the locusts mainly came from Eshan with a small portion from Hongta. The trajectory simulation results exhibit strong concordance with empirical field observations. Specifically, the backward-traced endpoints for locust swarms documented in Ning’er and Mojiang County during early July were geolocated in Jiangcheng County, which was temporally consistent with the confirmed large-scale influx of *C. kiangsu* into this area in late June. Furthermore, the backward trajectory analyses for infestations recorded in Yuanjiang and Xinping County during mid-to-late July revealed Mojiang and Jiangcheng County as probable origins, which was preceded by a distinct migratory peak that had infiltrated multiple districts of Pu’er City. The spatiotemporal distribution of the trajectory endpoints aligns with the peak migration period of *C. kiangsu*. This analysis demonstrates that the early-stage locust populations in Yunnan Province primarily originated from Phongsaly, Laos. After entering Pu’er, some locusts migrated further north and west, reaching areas such as Yuxi and Chuxiong.

### 3.3. Trajectory and Source Regions in Peak Migratory Period

**Figure 4:**
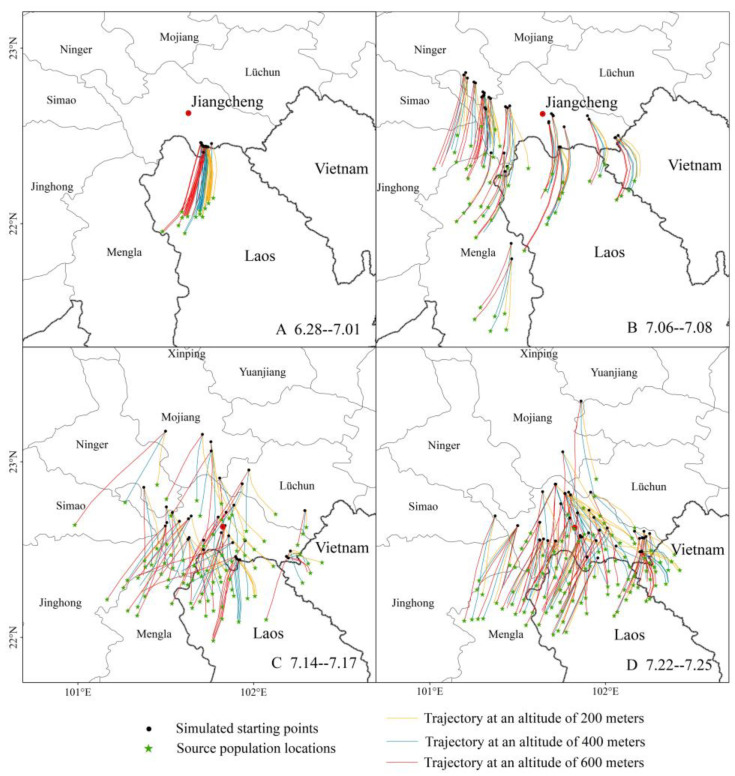
Backward trajectories of *C. kiangsu* during the peak migration period in 2020. (**A**–**D**) Description of the four migration peaks, respectively. The green star represents source population locations of *C. kiangsu*.

According to the results of the immigration hotspot analysis of *C. kiangsu* populations and the migration scale monitored by insect radar, a small number of *C. kiangsu* crossed the border from Laos and landed near the border in 2023. Therefore, the study of migration trajectories focused on 2020. During 2020, the peak immigration period of *C. kiangsu* from overseas was divided into four intervals, corresponding to four migration peaks: late June (28–30 June), early July (6–8 July), mid-July (14–17 July), and late July (22–25 July). Backward trajectory simulations of the *C. kiangsu* were conducted. By integrating the Chinese vegetation dataset with Google Earth satellite imagery, we identified valid trajectory landing points during the migration peak period, which correspond to the species’ source population locations. The simulation results and source population distribution are shown in Figure 4.

In late June, the backward trajectories of *C. kiangsu* migrating to Menglie Township, Jiangcheng County, at different altitudes consistently converged in Phongsaly, Laos (Figure 4A). In early July, the hotspot distribution areas of *C. kiangsu* covered most of Jiangcheng County, southern Ning’er County, and parts of Mengla County. Representative sites were selected for backward trajectory analysis. The trajectories from southern Ning’er predominantly terminated in Zhengdong Township, Jiangcheng County with a minority extending to Xishuangbanna. However, no *C. kiangsu* were detected in Xishuangbanna during early July, suggesting that the local populations likely originated from dispersal near Jiangcheng County. The backward trajectories in Mengla County remained within its boundaries. Given the established populations of *C. kiangsu* in Mengla County [12], it is hypothesized that the populations there derived partially from Phongsaly, Laos, and partially from local sources. In Jiangcheng County, most trajectories terminated in Phongsaly, Laos, while the western Zhengdong Township received partial sources from Mengla County. In the eastern Qushui Township, the trajectories at 200 m altitude partially terminated in Dien Bien Province, Vietnam, whereas trajectories at 400 m and 600 m altitudes crossed the Vietnam–Laos border into Phongsaly, Laos (Figure 4B).

By mid-July, the hotspot areas of migrating *C. kiangsu* expanded northward, covering all of Jiangcheng and Ning’er, southern Mojiang, and southwestern Lüchun counties. The backward trajectories from southern Ning’er County indicated origins in Kangping and Zhengdong Township, Jiangcheng. The trajectories from southern Mojiang County clustered in Jiangcheng County and the China–Laos border area. Trajectories from western Lüchun County terminated in Jiahe Township, Jiangcheng, while southern trajectories originated from Qushui Township, Jiangcheng. The trajectories in Jiangcheng County revealed that most sources originated from Phongsaly, Laos with partial sources in eastern Qushui Township from Dien Bien, Vietnam. At 400 m and 600 m altitudes in western Kangping and Zhengdong Township, the trajectories suggested potential origins in Xishuangbanna. Thus, the mid-July migration peak predominantly originated from Phongsaly, Laos, with minimal contributions from Xishuangbanna and Vietnam (Figure 4C).

In late July, the final migration peak of *C. kiangsu* occurred with hotspots concentrated in Jiangcheng County and sporadic influxes into Mojiang and Yuanjiang counties. The backward trajectories for sporadic influxes in these two areas primarily terminated in Jiangcheng County. In Jiangcheng, most trajectories ended in Phongsaly, Laos, except for isolated western trajectories terminating in Xishuangbanna and eastern Qushui Township trajectories in Dien Bien, Vietnam (Figure 4D).

In summary, the source distribution of *C. kiangsu* invading Yunnan exhibited pronounced spatial aggregation. During late June and early July, the primary sources originated from Laos with minor contributions from Xishuangbanna. From mid- to late July, populations in areas north of Jiangcheng County derived from the dispersal of previously migrated locusts, while the majority of Jiangcheng County’s sources remained concentrated in Laos, which was supplemented by limited inputs from Vietnam.

### 3.4. Forward Trajectory of Remigration C. kiangsu in Yunnan

A spatiotemporal analysis of *C. kiangsu* migration peaks and source region clustering identified mid-to-late July as the mass migration phase with primary landing clusters concentrated in Jiangcheng and Mojiang counties. Since Mojiang County populations derived from either Jiangcheng County or transboundary zones along the China–Laos border, the representative infestation points in Mojiang County were selected as simulation origins for remigration trajectory modeling. Simulations were initiated two days following the termination of the third migratory surge.

**Figure 5:**
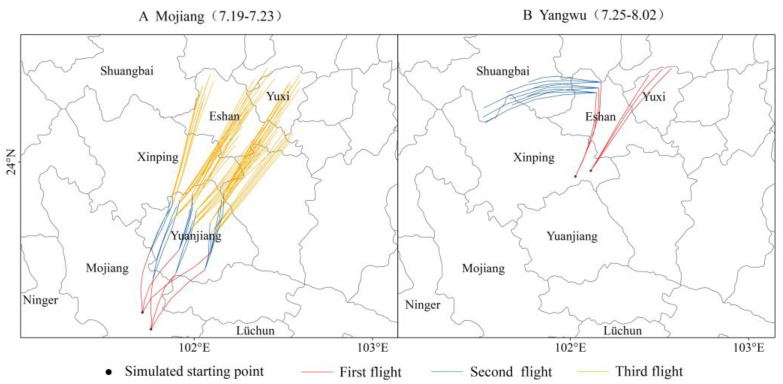
The forward trajectories of the remigration *C. kiangsu* in 2020.

The forward trajectory modeling predicted that *C. kiangsu* would reach the northernmost regions of Eshan County and Hongta District after three successive migration events (Figure 5A). Notably, isolated discrepancies between the simulation outcomes and field observations were identified. Specifically, among the 235 monitoring points collected in 2020, we filtered the migration points for the relevant time periods and found that more points were located in Yangwu Town, Xinping County, rather than in the simulated trajectory endpoint areas. This divergence was driven by precipitation-induced aggregation and partial settlement in Yangwu with remaining populations dispersing northward to Eshan County and Hongta District. Consequently, Yangwu was designated as new starting points for remigration trajectory modeling. The results demonstrated that under conducive meteorological conditions, *C. kiangsu* cohorts originating from Yangwu could migrate westward in early August, traversing Eshan County to invade Shuangbai County (Figure 5B). Subsequent meteorological shifts toward persistent northeasterly airflow patterns suppressed any further northward migration of *C. kiangsu*. This finding corresponds with the monitoring data for *C. kiangsu* in Shuangbai County during early August, as presented in Table 2.

The simulation results for Eshan County and Hongta District in Figure 5B indicated the presence of *C. kiangsu* by late July, whereas the field surveys documented in Table 2 first detected the species in early August. We hypothesized that this temporal mismatch likely arose from constraints (e.g., sampling intervals or spatial coverage limitations) during the field investigations, thereby delaying the detection of the initial migration events.

### 3.5. Wind Field Analysis During C. kiangsu Migration

The airflow analysis demonstrates that from June to July, a sustained southwesterly airflow has penetrated the 850 hPa level across southwestern China and adjacent regions, persisting with a northward progression trajectory. Under this synoptic configuration, 850 hPa winds over Laos and Yunnan Province exhibited consistent southwesterly orientations in late June. The winds progressively shifted to south–southwesterly directions throughout July. This evolving atmospheric regime established persistent wind corridors, thus facilitating the migration of *C. kiangsu* populations during their northward migration phase.

**Figure 6:**
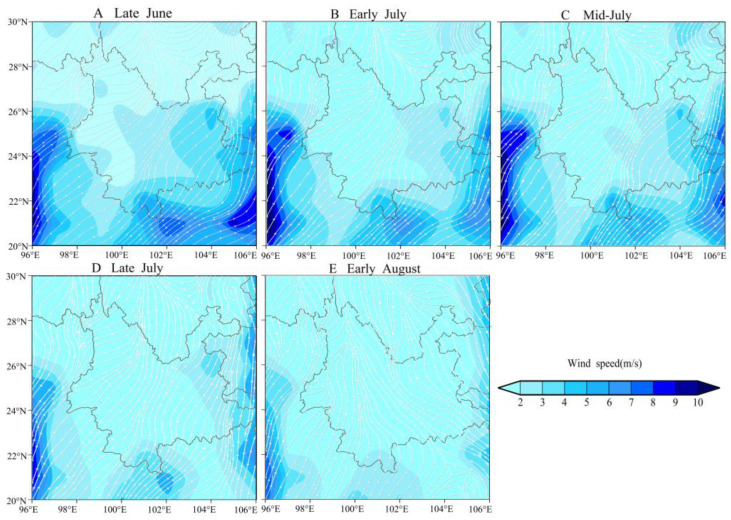
Average wind field at 850 hPa from late June to early August in 2020. The white arrow shows the wind direction. The blue color shows the wind speed.

Beginning in August, southeasterly–southerly winds prevailed over Laos and Yunnan. This mesoscale circulation pattern redirected the remigration of *C. kiangsu* populations northwestward, thereby providing infestation sources to northern Hongta and Chuxiong (Figure 6E). Notably in the Sino-Vietnamese border area, the air currents entering Yunnan Province from the Vietnamese sector are fundamentally sourced from Laos. This meteorological evidence suggests that *C. kiangsu* populations invading Qushui Township, Jiangcheng County via Vietnam likely originated from Laos.

### 3.6. Migration Corridors of C. kiangsu

**Figure 7:**
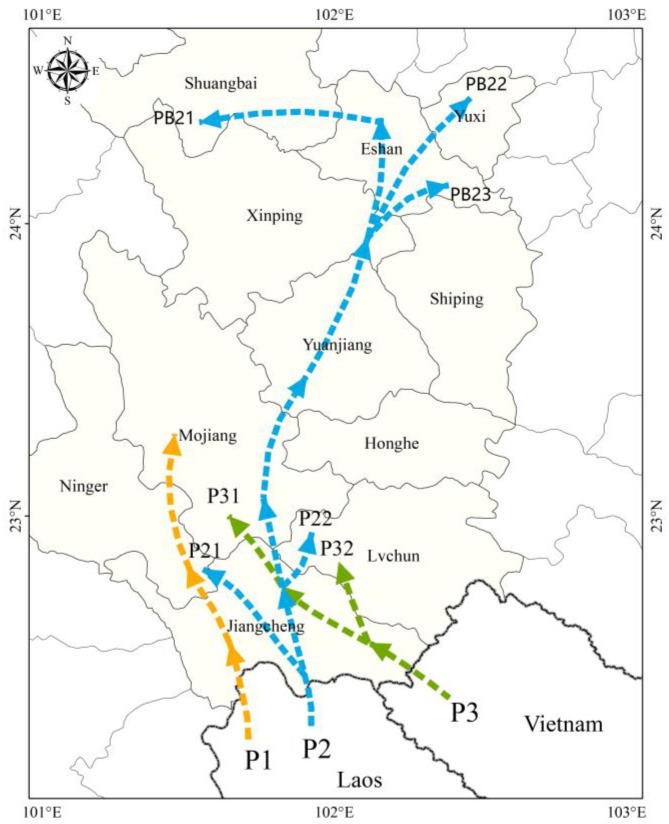
The transboundary migration corridors of *C. kiangsu*. P1: The Laos–Mengkang Port corridor. P2: Laos–Niuluohe border corridor, P21 is branch 1 of the second corridor, P22 is branch 2 of the second corridor. PB21, PB22, PB23 were the regions that *C. kiangsu* reached on the main migration axis. P3: Vietnam–Qushui corridor, P31 and P32 were the regions that *C. kiangsu* reached on the third corridor.

Based on an integrated analysis of *C. kiangsu’s* migration trajectories, the meteorological patterns during dispersal periods, and source tracing studies (including the initial migration time and peak migration intensity), three transboundary migration corridors into Yunnan Province were identified (Figure 7).

Corridor 1 (Laos–Mengkang Port): During early to mid-July, southwest airflow-mediated transport facilitated locust migration from Laos through Mengkang Port → Jiangcheng → Ning’er → the convergence zone southeast of the Ailao Mountains.

Corridor 2 (Laos–Niuluohe): From late June to early August, the main migration axis followed southerly airflow trajectories: Niuluohe Port → Jiangcheng → Mojiang → Yuanjiang → Xinping → Eshan/Hongta → Shuangbai. Branch 1: Northward dispersal from Jiangcheng to Ning’er County; Branch 2: Mid-July southwest airflow-mediated transport to Lüchun.

Corridor 3 (Vietnam–Qushui): From early to late July, southwest–southerly airflow transport enabled migratory populations from Vietnam → southeastern Jiangcheng → partial dispersal to Lüchun County. The Laos–Niuluohe border corridor, exhibiting prolonged migration duration and peak flux density, constituted the principal migration route.

## 4. Discussion

Since 2018, *C. kiangsu* has successively migrated across the China–Laos border into Yunnan, primarily concentrating in Jiangcheng County with smaller distributions being observed in Mengla and Mojiang counties. Bordering the Indochinese Peninsula, Jiangcheng County serves as the first stopover for the northward monsoon-driven migration of these locusts. This pattern highlights exceptionally close insect source exchanges between southern Yunnan and northern Laos [32], as the Indochinese Peninsula—characterized by a tropical climate, diverse plant species, and complex agricultural systems—functions as a year-round breeding ground for many critical agricultural and forestry pests, earning it the designation of a “perennial insect reservoir”. Furthermore, these low-elevation regions with higher temperatures support extensive growth of bamboo, corn, and rice—key host plants for *C. kiangsu*. The favorable habitat provides ample food resources and oviposition sites, meaning that large populations of cross-border migratory locusts typically undertake secondary migrations only when optimal weather conditions coincide.

Research has identified the Niuluohe corridor as the primary migratory route for *C. kiangsu*. Monitoring data from consecutive years reveal that both the early and peak migration phases of *C. kiangsu* involve cross-border entry into China through this corridor. A critical question arises: Why does the Niuluohe corridor dominate the entire migration cycle of *C. kiangsu*? Is this due to its valley terrain facilitating locust movement? Studies on *S. frugiperda* indicate that river valleys often act as migration corridors for its populations while also exhibiting barrier and convergence effects on their dispersal [33]. This raises further questions: Does valley terrain exert similar or analogous influences on *C. kiangsu*? If so, how do such topographic features shape insects’ decision-making during migration? These mechanisms represent key priorities for future research.

We assumed that *C. kiangsu* migrates passively with wind currents without accounting for its intrinsic flight speed or responses to varying atmospheric conditions. However, insects with autonomous flight capabilities are not entirely passive in aerial displacement; they exhibit active behavioral modulation [18]. Medium-to-large insects, such as locusts, dragonflies, and noctuid moths, demonstrate collective orientation and stratified aggregation behaviors. These strategies enable rapid and efficient migration, enhance migration success rates, and represent adaptive ecological strategies [34,35,36]. Notably, few studies have scientifically measured the flight speed of *C. kiangsu* or clarified its in-flight behavioral strategies. Furthermore, the HYSPLIT model does not incorporate biological flight behaviors (e.g., orientation and airspeed) when simulating insect migration trajectories [37]. This limitation may compromise the accuracy of the simulated migratory pathways. In the present study, the synergistic effects of wind direction, wind speed, and the self-powered flight speed of *C. kiangsu* could not be fully characterized, owing to both the absence of empirical flight speed data and inherent model constraints. Consequently, actual migration distances are likely to exceed simulated estimates. Future research will prioritize the quantification of the species’ flight kinematics to refine trajectory simulation precision.

Insect flight initiation is influenced by multiple factors, particularly the temperature [38]. Migratory insects typically initiate mass takeoffs under thermally suitable conditions, and airborne swarms actively select altitudes with temperatures meeting their flight threshold [39,40]. When ambient temperatures fall below this threshold, swarms either adjust their flight altitude or descend [41]. For example, *Locusta migratoria* are forced to land at temperatures below 19 °C [42], while *Oedaleus asiaticus* exhibits optimal survival at 20–25 °C and demonstrates enhanced flight capacity between 16 and 28 °C [43]. Although *C. kiangsu* belongs to the same taxonomic group, its flight threshold temperature remains uncharacterized. Laboratory experiments indicate that *C. kiangsu* achieves peak survival rates at 30 °C, suggesting that its thermal adaptation exceeds that of *O. asiaticus* [44]. Do these findings imply that *C. kiangsu* possesses a higher flight threshold temperature compared with other locust species? To address these questions, we will conduct systematic experiments to quantify the intrinsic flight speed, thermal flight range, and abiotic drivers of migration in *C. kiangsu*. The further research aims to elucidate the behavioral patterns and environmental triggers underlying its migratory behavior, thereby advancing a precise mechanistic understanding of its flight ecology.

As a natural disaster, locust plagues exhibit certain cyclical patterns in their formation and occurrence. For instance, *Locusta migratoria manilensis* shows outbreak intervals ranging from 9–11 years at the longest to 4–5 years at the shortest [45]. Since the 1990s, four major outbreaks of this subspecies have been recorded in Hebei Province, China [46]. However, the periodicity of locust plagues is not isolated; it is closely linked to factors such as drought and the overexploitation of land, which create intrinsic causal relationships [11]. These factors also contribute to the synchronized, large-scale emergence of locust swarms. Yunnan experienced its first migration of *C. kiangsu* from neighboring countries in 2018. Subsequent sporadic border crossings occurred in 2019, 2021, and 2022 with a massive migratory influx in 2020 causing significant agricultural damage. Small-scale populations were detected in 2023, but no incursions have been observed as of 2022. Meteorological analysis suggests that the early spring drought and high temperatures in Laos during 2020 accelerated egg hatching, improved survival rates, and advanced migration timelines, creating optimal conditions for the early and large-scale migration. Beyond climatic drought, habitat alterations due to land-use changes also contributed to these outbreaks.

Global climate change is becoming increasingly frequent with a rise in extreme weather events. Studies indicate that under the context of global warming, particularly the rising winter temperatures, the overwintering eggs of locusts are more likely to survive, providing a “seed bank” for outbreaks of locust infestations in the following year. Additionally, the compounding effects of intensified droughts, pasture degradation, and other factors create suitable breeding grounds for locusts to lay eggs, coupled with their strong adaptability to arid conditions. Consequently, due to expanding habitats, phenological changes, and migratory behaviors, the frequency and scale of *C. kiangsu* outbreaks may escalate significantly in the future [47]. Therefore, clarifying the migration timing and pathways of *C. kiangsu* holds critical importance for implementing targeted control measures across regions and timeframes, thereby enhancing pest management efficiency.

## 5. Conclusions

Our findings demonstrate that Laos constitutes the principal source region for the transboundary migrations of *C. kiangsu*. Populations initially dispersed from Laos are hypothesized to traverse Vietnamese airspace via atmospheric currents before infiltrating Yunnan Province, underscoring the China–Laos border as a critical focal area for *C. kiangsu* control. Following the 2018 migration, small established populations were observed in Mengla County [12], providing localized sources for subsequent domestic migration. In addition to the three transboundary migration corridors, a fourth domestic route was identified. Furthermore, airflow analysis suggests that Mengla County may become a potential high risk deposition zone for cross-border *C. kiangsu* migrations in August.

Meteorological forcing emerges as the principal extrinsic factor governing *C. kiangsu* dispersal dynamics. In July, prevailing southwestern winds over southern Yunnan facilitate the sustained northward migration of populations from the Indochina Peninsula and southern Yunnan, enabling locusts to reach Yuxi City. However, the shift to southeastern winds in early August partially disrupts these northward movements. Precipitation forces *C. kiangsu* to land, with locusts preferentially selecting habitats dominated by preferred host plants, such as bamboo and maize. Trajectory analysis of *C. kiangsu* cohorts departing Mojiang County revealed an initial dispersal toward Eshan County and the Hongta District northern periphery. However, mid-flight interception by precipitation systems resulted in a forced deposition at Yangwu Township, Xinping County. The subsequent resumption of westerly migration toward Chuxiong Prefecture under improved meteorological conditions was temporally congruent with the field surveillance data, empirically validating precipitation as a critical modulator of depositional phase transitions in *C. kiangsu* migratory cycles.

## Figures and Tables

**Table 1:** The damage degree of woodland and cropland caused by *C. kiangsu*.

Damage Degree	Characterization	
	Woodland	Cropland
Mild Damage	Less than 1/3 of the host leaves are eaten	1–10% of affected plants
Moderate Damage	1/3–2/3 of host leaves are eaten	11–40% of affected plants
Severe Damage	More than 2/3 of host leaves are eaten	More than 41% of affected plants

**Table 2:** Locations and first appearance dates of adults migrating in Yunnan during 2020.

State (City)	County (District)	Township	Longitude and Latitude	Adult First Appearance Date
Pu’er	Jiangcheng	Menglie	101.896, 22.461	06-28
	Ning’er	Liming	101.492, 22.753	07-06
	Simao	Yixiang	101.039, 22.708	07-07
	Mojiang	Wenwu	101.631, 23.023	07-08
Xishuangbanna	Mengla	Mengyang	101.649, 21.804	07-03
Honghe	Lüchun	Daheisan	101.921, 22.876	07-14
Yuxi	Yuanjiang	Inyuan	101.861, 23.352	07-15
	Xinping	Yangwu	101.964, 23.892	07-24
	Xinping	Jianxing	101.683, 23.682	07-24
	Eshan	Zhennian	102.142, 24.166	08-06
	Hongta	Daying	102.427, 24.244	08-03
Chuxiong	Shangbai	Ainishan	101.577, 24.425	08-02

## Data Availability

The data presented in this study are available on request from the corresponding author due to restrictions involving specific geographical names and location information in border areas.
